# PMHT Approach for Multi-Target Multi-Sensor Sonar Tracking in Clutter

**DOI:** 10.3390/s151128177

**Published:** 2015-11-06

**Authors:** Xiaohua Li, Yaan Li, Jing Yu, Xiao Chen, Miao Dai

**Affiliations:** School of Marine Science and Technology, Northwestern Polytechnical University, Xi’an 710072, China; E-Mails: liyaan@nwpu.edu.cn (Y.L.); yujing@nwpu.edu.cn (J.Y.); chenxiao@mail.nwpu.edu.cn (X.C.); ly_dm@163.com (M.D.)

**Keywords:** probabilistic multi-hypothesis tracker (PMHT), multi-target multi-sensor sonar tracking, extended Kalman filter (EKF), unscented Kalman filter (UKF), data association

## Abstract

Multi-sensor sonar tracking has many advantages, such as the potential to reduce the overall measurement uncertainty and the possibility to hide the receiver. However, the use of multi-target multi-sensor sonar tracking is challenging because of the complexity of the underwater environment, especially the low target detection probability and extremely large number of false alarms caused by reverberation. In this work, to solve the problem of multi-target multi-sensor sonar tracking in the presence of clutter, a novel probabilistic multi-hypothesis tracker (PMHT) approach based on the extended Kalman filter (EKF) and unscented Kalman filter (UKF) is proposed. The PMHT can efficiently handle the unknown measurements-to-targets and measurements-to-transmitters data association ambiguity. The EKF and UKF are used to deal with the high degree of nonlinearity in the measurement model. The simulation results show that the proposed algorithm can improve the target tracking performance in a cluttered environment greatly, and its computational load is low.

## 1. Introduction

The active sonar system [1] is one of the most used sonar systems; it uses one or more transmitters and receivers. Transmitters emit acoustic signals, and receivers listen to echoes of this signal from a target. When the transmitter and receiver are co-located, it is described as monostatic; when the transmitter and receiver are not co-located, it is described as bistatic; when multiple transmitters or receivers are used and spatially separated, it is described as multistatic. Multistatic target tracking has attracted broad attention since the formation of the NATO Multistatic Tracking Working Group in 2005. The multistatic sonar system provides the following measurements: bistatic range, *i.e.*, time-of-arrival, bearing measurements, bistatic range-rate measurements if continuous waveform (CW) is used and signal-to-noise ratio (SNR). Different waveforms can yield different measurement types and uncertainties. For example, frequency-modulated (FM) signals provide more accurate range measurements, while CW signals deliver Doppler measurements in addition to lower resolution range measurements. Both the FM and CW signals deliver bearing measurements. Additionally, FM and CW signals can be transmitted simultaneously. In this paper, we consider only bistatic range and Doppler information.

The major advantage of the multistatic sonar system is that it has additional detection opportunities in comparison to a monostatic system. It is very hard for a target to remain covert in a multistatic sonar system due to the transmitter-target-receiver geometry. In addition, the multistatic sonar system also has the potential to reduce the overall measurement uncertainty. What is more, receivers can remain covert if they are spatially separated from the transmitter.

Despite the advantages of multistatic sonar tracking systems, their use is challenging because of the complexity of the underwater environment. The underwater environment is time varying and spare varying, characterized by low target detection probability and an extremely large number of false alarms caused by reverberation [2,3]. Meanwhile, the low speed of acoustic propagation might result in measurement time delay, which will increase uncertainty in target location.

The multistatic system requires some form of data association approach to associate estimates. Some of the common data association approaches used for multi-target tracking are: (1) nearest neighbor (NN)-based trackers [4]; (2) multiple hypothesis trackers (MHT) [5] and their variants, such as distributed MHT [6] and probabilistic MHT (PMHT) [7]; (3) a joint probabilistic data association filter (JPDA) [8]; (4) batch processing approaches, such as maximum likelihood probabilistic data association (MLPDA) [9,10] and maximum likelihood probabilistic multi-hypothesis trackers (ML-PMHT) [11,12,13]; (5) a probability hypothesis density (PHD) tracker [14] and its variants, such as Gaussian mixture cardinalized PHD (GMCPHD) [3,15,16]; (6) particle filtering (PF) and some of its variants [17,18]; and (7) a specular-cued tracker [19].

The PMHT is a natural and effective data association approach. The key idea is that it allows multiple measurements to be assigned to the same target/transmitter, which differs from the traditional data association approaches, such as JPDA [8]. Moreover, the computational burden of PMHT grows linearly with the number of targets detected. Furthermore, it works directly in the original Cartesian space, which reduces the amount of tuning. However, the “usual” PMHT algorithm just considers the data association ambiguity between measurements and targets [20,21,22]. For multistatic sonar target tracking, there is an additional unknown data association between measurements and transmitters, *i.e.*, we do not know which measurement arises from which transmitter, which increases data association complexity.

In this paper, we propose a novel PMHT tracker based on the extended Kalman filter (EKF) and unscented Kalman filter (UKF) for the problem of multi-target multi-sensor sonar tracking in a cluttered environment. In contrast to the “usual” PMHT, the proposed PMHT algorithm can handle both the measurement-to-target and measurement-to-transmitter association. In addition, we present a more compact formulation for the EKF-based PMHT, which obviates the need to form “stacked” measurements.

The remainder of this paper is as follows. Section 2 describes the system and measurement model for multi-target multi-sensor sonar tracking. The PMHT algorithm is provided in Section 3. Section 4 discusses the simulation results. Finally, Section 5 summarizes the paper and describes further work.

## 2. Problem Description

### 2.1. System Model

We assume that there are M targets in the two-dimensional (2D) Cartesian space. The number of targets is constant and known during the whole scan. The state vector of the *m*th target at time *t* is $x_{m}\left(t\right)={\left(x_{m}\left(t\right)\;{\dot{x}}_{m}\left(t\right)\;y_{m}\left(t\right)\;{\dot{y}}_{m}\left(t\right)\right)}^{T}$, where the target location is $p_{m}\left(t\right)={\left(x_{m}\left(t\right)\;y_{m}\left(t\right)\right)}^{T}$ and the target velocity is $v_{m}\left(t\right)={\left({\dot{x}}_{m}\left(t\right)\;{\dot{y}}_{m}\left(t\right)\right)}^{T}$.

For the underwater target tracking, we assume the target states satisfy a nearly constant velocity model [23]:

(1) $$x_{m}\left(t\right)=Fx_{m}\left(t-1\right)+v_{m}\left(t\right)$$

where $F=I_{2}⊗\tilde{F}$ is the system state transition matrix, $\tilde{F}=\left(\begin{array}{cc} 1 & \Delta T\\ 0 & 1 \end{array}\right)$, ΔT is the sampling interval, ⊗ is the Kronecker product and $I_{2}$ is the two-dimensional identity matrix. The system is corrupted with zero-mean white Gaussian process noise $v_{m}\left(t\right)$ with covariance matrix $Q_{m}=\sigma_{p}^{2}I_{2}⊗\tilde{Q}$, where $\sigma_{p}^{2}$ is the process noise intensity, and $\tilde{Q}=\left(\begin{array}{cc} \frac{\Delta T^{4}}{4} & \frac{\Delta T^{2}}{2}\\ \frac{\Delta T^{2}}{2} & \Delta T^{2} \end{array}\right)$.

### 2.2. Measurement Model

**Figure 1:**
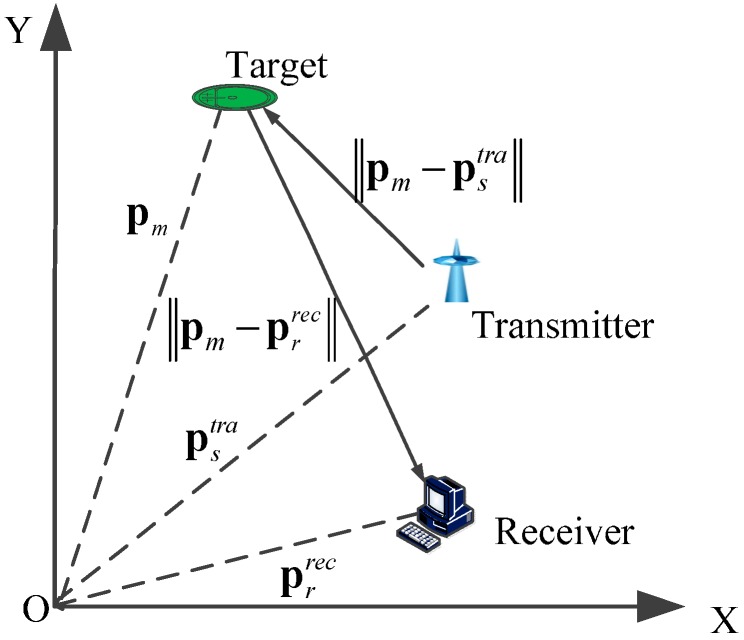
Target geometry for multi-target multi-sensor tracking.

We assume the multistatic sonar system consists of S transmitters and R receivers, and the transmitters and receivers are stationary and their positions known. Let $p_{r}^{rec}={\left(x_{r}^{rec}\;y_{r}^{rec}\right)}^{T}\left(r=1,2,\cdots ,R\right)$ be the receivers’ location and $p_{s}^{tra}={\left(x_{s}^{tra}\;y_{s}^{tra}\right)}^{T}\left(s=1,2,\cdots ,S\right)$ be the transmitters’ location. An overview of the setting is shown in Figure 1.

In Figure 1, $\left\|p_{m}\left(t\right)-p_{s}^{tra}\right\|$ is the distance between target m and transmitter s, and $\left\|p_{m}\left(t\right)-p_{r}^{rec}\right\|$ is the distance between target m and receiver r. Different from Coralippi’s paper [24], here we consider measurements of bistatic range (delay) $\gamma \left(x_{m}\left(t\right)\right)$ and Doppler $D\left(x_{m}\left(t\right)\right)$. The acquired measurement $z_{n}\left(t\right)$ is described as follows,

(2) $$z_{n}\left(t\right)=h\left(x_{m}\left(t\right),p_{s}^{tra}\right)+w_{n}\left(t\right)\text{=}\left[\begin{array}{c} \gamma \left(x_{m}\left(t\right)\right)\\ D\left(x_{m}\left(t\right)\right) \end{array}\right]+w_{n}\left(t\right)\;$$

where $h\left(x_{m}\left(t\right),p_{s}^{tra}\right)$ is the measurement function and $w_{n}\left(t\right)$ is independent zero-mean white Gaussian noise with covariance matrix $R_{n}\left(t\right)$,

(3) $$R_{n}\left(t\right)=\left[\begin{array}{cc} \sigma_{r}^{2} & 0\\ 0 & \sigma_{D}^{2} \end{array}\right]$$

in which $\sigma_{r}^{2}$ and $\sigma_{D}^{2}$ denote the variances of the range and Doppler, respectively.

The range and Doppler measurement [19,25] for target m and transmitter s are given by:

(4a) $$\gamma \left(x_{m}\left(t\right)\right)=\left\|p_{m}\left(t\right)-p_{s}^{tra}\right\|+\left\|p_{m}\left(t\right)-p_{r}^{rec}\right\|$$

(4b) $$D\left(x_{m}\left(t\right)\right)=\left[\frac{{\left(p_{m}\left(t\right)-p_{r}^{rec}\right)}^{T}}{\left\|p_{m}\left(t\right)-p_{r}^{rec}\right\|}+\frac{{\left(p_{m}\left(t\right)-p_{s}^{tra}\right)}^{T}}{\left\|p_{m}\left(t\right)-p_{s}^{tra}\right\|}\right]\cdot \left(-\frac{f}{c}\right)\cdot v_{m}\left(t\right)$$

in which f is the working frequency of the transmitter, c is the speed of sound in water and $v_{m}\left(t\right)$ is the velocity of the target m.

As the linear Kalman filter cannot handle the nonlinear measurements in the range and Doppler, in this work, we use the EKF [26] and UKF to update the target state. For the EKF, we linearize the nonlinear measurement function $h\left(x_{m}\left(t\right),p_{s}^{tra}\right)$. As opposed to the EKF, the UKF does not approximate the nonlinear measurement function. Instead, based on the unscented transform (UT) [27], the UKF uses a deterministic sampling approach (sigma-points) to capture the posterior distribution mean and covariance. From now on, we call the EKF-based PMHT the PMHTe and UKF-based PMHT the PMHTu.

## 3. The PMHT Approach with Unknown Transmitter Association

In this section, we derive the PMHT method for multi-target multi-sensor sonar tracking with unknown measurement-to-target and measurement-to-transmitter association. We assume all measurements’ qualities for a particular transmitter are different and use the sequential updating for the UKF smoother.

### 3.1. Notation

Let there be M targets and $N_{t}$ measurements for a receiver at time t. The measurements consist of the detected target returns and clutter measurements. As a result, the collections of states for all targets and available measurements at time t are written as $X_{t}=\left(x_{1}\left(t\right),\cdots ,x_{M}\left(t\right)\right)$ and $Z_{t}=\left(z_{1}\left(t\right),\cdots ,z_{N_{t}}\left(t\right)\right)$. The collection of states and measurements for all targets up to time T are $X=\left(X_{1},X_{2},\cdots ,X_{T}\right)$ and $Z=\left(Z_{1},Z_{2},\cdots ,Z_{T}\right)$. Now, our goal is to estimate the target states X.

In the “usual” PMHT, we write $a_{n}\left(t\right)=m$ if measurement n is associated with a target m. Similarly, here, we define $b_{n}\left(t\right)=s$ if measurement n is associated with transmitter s. Then, the prior probabilities’ associations are denoted as:

(5a) $$p\left(a_{n}\left(t\right)=m\right)=\pi_{m}^{a}$$

(5b) $$p\left(b_{n}\left(t\right)=s\right)=\pi_{s}^{b}$$

The statistically-independent measurement-to-target and measurement-to-transmitter associations at time t are $A_{t}=\left(a_{1}\left(t\right),a_{2}\left(t\right),\cdots ,a_{N_{t}}\left(t\right)\right)$ and $B_{t}=\left(b_{1}\left(t\right),b_{2}\left(t\right),\cdots ,b_{N_{t}}\left(t\right)\right)$, respectively. The associations for the entire batch up to time T are $A=\left(A_{1},A_{2},\cdots ,A_{T}\right)$ and $B=\left(B_{1},B_{2},\cdots ,B_{T}\right)$.

### 3.2. The PMHTu Algorithm

PMHT is an expectation maximization (EM)-based [28], batch-tracking algorithm for multi-target tracking in a cluttered environment. The aim of the PMHT approach is to maximize p(X|Z) over X using the EM algorithm. That is, we should find the maximum *a posteriori* (MAP) estimate of X

(6) $${\hat{X}}_{MAP}=\arg\underset{\text{X}}{\max}\text{ E}\left\{\ln\left(p\left(X|Z\right)\right)\right\}$$

Usually, it is difficult to directly evaluate the MAP expectation. Hence, we define the following function:

(7) $$Q\left(X^{\left(l+1\right)};X^{\left(l\right)}\right)=\int_{\text{A},\text{B}}\ln\left(p\left(X^{\left(l+1\right)},A,B|Z\right)\right)\cdot p\left(A,B|X^{\left(l\right)}\text{,}Z\right)$$

where l is the number of the EM iterative steps. Based on an initial estimate $X^{\left(0\right)}$, the PMHT aims to find:

(8) $$X^{\left(l+1\right)}=\arg\underset{X^{\left(l+1\right)}}{\max}\;Q\left(X^{\left(l+1\right)};X^{\left(l\right)}\right)$$

until a desired degree of convergence is achieved.

In order to derive an analytic expression for $Q\left(X^{\left(l+1\right)};X^{\left(l\right)}\right)$, we note that the posterior association probabilities for all measurements in Equation (7) can be written as:

(9) $$p\left(A,B\text{|}X^{\left(l\right)}\text{,}Z\right)=\sum_{t=1}^{T}\sum_{n=1}^{N_{t}}w_{m,n}^{\left(l\right)}\left(t,s\right)$$

where:

(10) $$w_{m,n}^{\left(l\right)}\left(t,s\right)=\frac{\pi_{m}^{a}\pi_{s}^{b}p\left(z_{n}\left(t\right)|x_{m}^{\left(l\right)}\left(t\right),a_{n}\left(t\right)=m,b_{n}\left(t\right)=s\right)}{\sum_{p=1}^{M}\sum_{q=1}^{S}\pi_{p}^{a}\pi_{q}^{b}p\left(z_{n}\left(t\right)|x_{p}^{\left(l\right)}\left(t\right),a_{n}\left(t\right)=p,b_{n}\left(t\right)=q\right)}$$

The derivation of $w_{m,n}^{\left(l\right)}\left(t,s\right)$ is given in Appendix A. Here, $w_{m,n}^{\left(l\right)}\left(t,s\right)$ denotes the posterior association probability of measurement n being related to target m and transmitter s at time t. λ is the spatial density of clutter, and V is the measurements’ region of volume. Assume the clutters are uniformly distributed in range and Doppler measurement space. Additionally, the number of clutter ε follows the Poisson distribution:

(11) $$p\left(\varepsilon \right)=\frac{{\left(\lambda V\right)}^{\varepsilon }}{\varepsilon !}e^{-\lambda V}$$

Now, we obtain (the detailed derivation is given in Appendix B):

(12) $$\begin{array}{l} Q\left(X^{\left(l+1\right)};X^{\left(l\right)}\right)=\sum_{A,B}\ln\left(p\left(X^{\left(l+1\right)},A,B\text{|}Z\right)\right)p\left(A,B|X^{\left(l\right)},Z\right)\\ =\ln\left(\prod_{m=1}^{M}p\left(x_{m}^{\left(l+1\right)}\left(1\right)\right)\prod_{t=2}^{T}\prod_{m=1}^{M}p\left(x_{m}^{\left(l+1\right)}\left(t\right)|x_{m}^{\left(l+1\right)}\left(t-1\right)\right)\right)\\ +\sum_{s=1}^{S}\sum_{t=1}^{T}\sum_{n=1}^{N_{t}}\sum_{m=1}^{M}(w_{m,n}^{\left(l\right)}\left(t,s\right)\log\left(\pi_{m}^{a}\pi_{s}^{b}\right)+w_{m,n}^{\left(l\right)}\left(t,s\right)\ln p\left(z_{n}\left(t\right)|x_{m}^{\left(l\right)}\left(t\right),a_{n}\left(t\right)=m,b_{n}\left(t\right)=s\right)) \end{array}$$

In order to maximize $Q\left(X^{\left(l+1\right)};X^{\left(l\right)}\right)$ in Equation (12), we should find its gradient. It turns out that Equation (12) has the same derivative as:

(13) $$\begin{array}{ll} {\hat{Q}}_{1}(X^{\left(l+1\right)};X^{\left(l\right)}) & =\ln\prod_{m=1}^{M}p\left(x_{m}^{\left(l+1\right)}\left(1\right)\right)\prod_{t=2}^{T}\prod_{m=1}^{M}p\left(x_{m}^{\left(l+1\right)}\left(t\right)|x_{m}^{\left(l+1\right)}\left(t-1\right)\right)\\ & -\frac{1}{2}\sum_{m=1}^{M}\sum_{s=1}^{S}\sum_{t=1}^{T}\left[\left({\tilde{z}}_{m,s}\left(t\right)-h\left(x_{m}^{\left(l+1\right)}\left(t\right),p_{s}^{tra}\right)\right){\tilde{R}}_{m,s}{\left(t\right)}^{-1}{\left({\tilde{z}}_{m,s}\left(t\right)-h\left(x_{m}^{\left(l+1\right)}\left(t\right),p_{s}^{tra}\right)\right)}^{T}\right] \end{array}$$

where the synthetic measurement ${\tilde{z}}_{m,s}\left(t\right)$ and covariance ${\tilde{R}}_{m,s}\left(t\right)$ are given by:

(14) $${\tilde{z}}_{m,s}\left(t\right)=\frac{\sum_{n=1}^{N_{t}}w_{m,n}^{\left(l\right)}\left(t,s\right)z_{n,s}\left(t\right)}{\sum_{n=1}^{N_{t}}w_{m,n}^{\left(l\right)}\left(t,s\right)}$$

(15) $${\tilde{R}}_{m,s}\left(t\right)=\frac{R_{m,s}\left(t\right)}{\sum_{n=1}^{N_{t}}w_{m,n}^{\left(l\right)}\left(t,s\right)}$$

There is no association uncertainty for maximizing the $Q\left(X^{\left(l+1\right)};X^{\left(l\right)}\right)$, so that we can employ the UKF smoother to solve the maximization problem. Here, Equation (13) reflects the nonlinearity of the measurement model in Equation (4).

There are two methods to update the Kalman filter for a multi-sensor scenario: sequential updating and parallel updating [29]. In sequential updating, the state vector and state covariance matrix are updated sequentially. The sequential updating can be easily extended to a larger number of sensors. In the parallel updating, we stack the measurements and update them in one step. Data from all sensors are expected; also, it is not easy to extend the number of sensors. An additional disadvantage of the parallel updating is that it needs to operate large measurement vectors and matrices, which is more computationally expensive. In this paper, we use the sequential updating method.

### 3.3. PMHTe Simplification for the Multi-Sensor Case

In this section, we present a compact formulation that obviates the need to form the “stacked” measurements for the PMHTe.

For the EKF, the derivatives of the range and Doppler observed by a transmitter s with respect to the target m are given by:

(16a) $$\frac{\partial \gamma }{dx_{m}}=\frac{x_{m}-x_{r}^{rec}}{\left\|p_{m}-p_{r}^{rec}\right\|}+\frac{x_{m}-x_{s}^{tra}}{\left\|p_{m}-p_{s}^{tra}\right\|}$$

(16b) $$\frac{\partial \gamma }{d{\dot{x}}_{m}}=0$$

(16c) $$\begin{array}{l} \frac{\partial D}{dx_{m}}=\frac{x_{m}}{\left\|p_{m}-p_{r}^{rec}\right\|}-\frac{\left(x_{m}-x_{r}^{rec}\right)\zeta_{r}^{rec}}{{\left\|p_{m}-p_{r}^{rec}\right\|}^{3}}+\frac{x_{m}}{\left\|p_{m}-p_{s}^{tra}\right\|}-\frac{\left(x_{m}-x_{s}^{tra}\right)\zeta_{s}^{tra}}{{\left\|p_{m}-p_{s}^{tra}\right\|}^{3}}\\ \; \end{array}$$

(16d) $$\frac{\partial D}{d{\dot{x}}_{m}}=\frac{x_{m}-x_{r}^{rec}}{\left\|p_{m}-p_{r}^{rec}\right\|}+\frac{x_{m}-x_{s}^{tra}}{\left\|p_{m}-p_{s}^{tra}\right\|}$$

in which $\zeta_{r}^{rec}=\left(\left(x_{m}-x_{r}^{rec}\right){\dot{x}}_{m}+\left(y_{m}-y_{r}^{rec}\right){\dot{y}}_{m}\right)$ and $\zeta_{s}^{tra}=\left(\left(x_{m}-x_{s}^{tra}\right){\dot{x}}_{m}+\left(y_{m}-y_{s}^{tra}\right){\dot{y}}_{m}\right)$.

The derivatives of the range and Doppler for $y_{m}$ is similar to $x_{m}$. Therefore, the gradient of the nonlinear measurement function $h\left(x_{m},p_{s}^{tra}\right)$ becomes:

(17) $$H_{m,s}=\nabla h\left(x_{m},p_{s}^{tra}\right)=\left[\begin{array}{cccc} \frac{\partial \gamma }{dx_{m}} & \frac{\partial \gamma }{d{\dot{x}}_{m}} & \frac{\partial \gamma }{dy_{m}} & \frac{\partial \gamma }{d{\dot{y}}_{m}}\\ \frac{\partial D}{dx_{m}} & \frac{\partial D}{d{\dot{x}}_{m}} & \frac{\partial D}{dy_{m}} & \frac{\partial D}{d{\dot{y}}_{m}} \end{array}\right]$$

Considering the nonlinear system model in Equation (4) and performing a linearization at $h\left(x_{m}^{\left(l+1\right)}\left(t\right),p_{s}^{tra}\right)$, we get:

(18) $$\begin{array}{ll} \nabla_{X^{\left(l+1\right)}}Q\left(X^{\left(l+1\right)};X^{\left(l\right)}\right)= & \nabla_{X^{\left(l+1\right)}}\left[\ln\prod_{m=1}^{M}p\left(x_{m}^{\left(l+1\right)}\left(1\right)\right)\prod_{t=2}^{T}\prod_{m=1}^{M}p\left(x_{m}^{\left(l+1\right)}\left(t\right)|x_{m}^{\left(l+1\right)}\left(t-1\right)\right)\right]\\ & -\frac{1}{2}\sum_{s=1}^{S}\sum_{t=1}^{T}\sum_{n=1}^{N_{t}}\sum_{m=1}^{M}[w_{m,n}^{\left(l\right)}\left(t,s\right)H_{m,s}^{T}R_{m,s}{\left(t\right)}^{-1}\left({\hat{z}}_{n}\left(t\right)-H_{m,s}x_{m}^{\left(l+1\right)}\left(t\right)\right)] \end{array}$$

Rearranging and factorizing Equation (18) yields:

(19) $$\begin{array}{l} \nabla_{X^{\left(l+1\right)}}Q\left(X^{\left(l+1\right)};X^{\left(l\right)}\right)=\nabla_{X^{\left(l+1\right)}}\left[\ln\prod_{m=1}^{M}p\left(x_{m}^{\left(l+1\right)}\left(1\right)\right)\prod_{t=2}^{T}\prod_{m=1}^{M}p\left(x_{m}^{\left(l+1\right)}\left(t\right)|x_{m}^{\left(l+1\right)}\left(t-1\right)\right)\right]\\ +\sum_{t=1}^{T}\sum_{m=1}^{M}[\left(\sum_{s=1}^{S}\sum_{n=1}^{N_{t}}w_{m,n}^{\left(l\right)}\left(t,s\right)\cdot H_{m,s}^{T}R_{m,s}{\left(t\right)}^{-1}H_{m,s}\right){(\left(\sum_{s=1}^{S}\sum_{n=1}^{N_{t}}w_{m,n}^{\left(l\right)}\left(t,s\right)\cdot H_{m,s}^{T}R_{m,s}{\left(t\right)}^{-1}H_{m,s}\right)}^{-1}\\ \;\cdot \left(\sum_{s=1}^{S}\sum_{n=1}^{N_{t}}w_{m,n}^{\left(l\right)}\left(t,s\right)\cdot H_{m,s}^{T}R_{m,s}{\left(t\right)}^{-1}{\hat{z}}_{n}\left(t\right)\right)-x_{m}^{\left(l+1\right)}\left(t\right))] \end{array}$$

Finally, it turns out that Equation (12) has the same derivative with $\nabla_{X^{\left(l+1\right)}}{\hat{Q}}_{2}\left(X^{\left(l+1\right)};X^{\left(l\right)}\right)$, where:

(20) $$\begin{array}{ll} {\hat{Q}}_{2}\left(X^{\left(l+1\right)};X^{\left(l\right)}\right) & =\ln\left(\prod_{m=1}^{M}p\left(x_{m}^{\left(l+1\right)}\left(1\right)\right)\prod_{t=2}^{T}\prod_{m=1}^{M}p\left(x_{m}^{\left(l+1\right)}\left(t\right)|x_{m}^{\left(l+1\right)}\left(t-1\right)\right)\right)\\ & -\frac{1}{2}\sum_{t=1}^{T}\sum_{m=1}^{M}\left[\left({\tilde{z}}_{m}\left(t\right)-x_{m}^{\left(l+1\right)}\left(t\right)\right){\tilde{R}}_{m}{\left(t\right)}^{-1}{\left({\tilde{z}}_{m}\left(t\right)-x_{m}^{\left(l+1\right)}\left(t\right)\right)}^{T}\right] \end{array}$$

in which the synthetic measurement ${\tilde{z}}_{m}\left(t\right)$ and synthetic measurement covariance ${\tilde{R}}_{m}\left(t\right)$ are given by:

(21) $${\tilde{z}}_{m}\left(t\right)={\tilde{R}}_{m}\left(t\right)\sum_{s=1}^{S}\sum_{n=1}^{N_{t}}w_{m,n}^{\left(l\right)}\left(t,s\right)H_{m,s}^{T}R_{m,s}\left(t\right){\hat{z}}_{n}\left(t\right)$$

(22) $${\tilde{R}}_{m}\left(t\right)={\left(\sum_{s=1}^{S}\sum_{n=1}^{N_{t}}w_{m,n}^{\left(l\right)}\left(t,s\right)H_{m,s}^{T}R_{m,s}{\left(t\right)}^{-1}H_{m,s}\right)}^{-1}$$

Maximizing the $Q\left(X^{\left(l+1\right)};X^{\left(l\right)}\right)$ thus coincides with the maximum of a single sensor system with no association uncertainty, so that we can easily use the EKF smoother to update the target state by the synthetic measurement ${\tilde{z}}_{m}\left(t\right)$ and covariance ${\tilde{R}}_{m}\left(t\right)$.

## 4. Simulation

**Figure 2:**
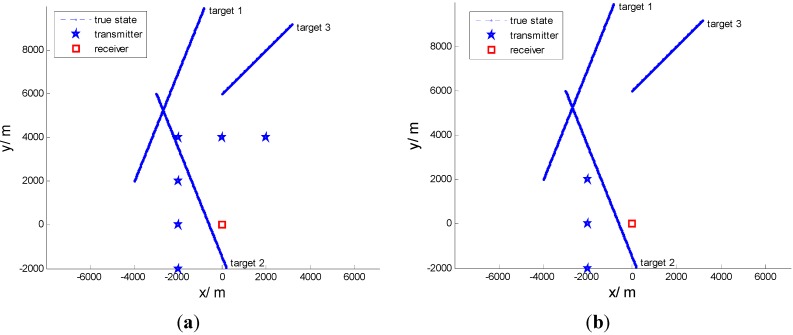
Three target tracks for 200 scans for one receiver: (**a**) Six transmitters; (**b**) Three transmitters.

The simulation consists of two scenarios. The first scenario consists of six transmitters and a single receiver. The second scenario consists of three transmitters and a single receiver. The transmitters and receiver are both stationary. Both scenarios treat the same three targets as seen in Figure 2. The targets’ parameters are shown in Table 1. The receiver is located at the origin of the ordinates. The six transmitters are placed at: (i) [−2000 m, −2000 m]; (ii) [−2000 m, 0 m]; (iii) [−2000 m, 2000 m]; (iv) [−2000 m, 4000 m]; (v) [0 m, 4000 m]; and (vi) [2000 m, 4000 m]. The frequency of the transmitters is 20 kHz. We assume the probability of detection is equal and constant for all targets; here, we set it to 0.5. The number of clutters is Poisson distributed with mean 60 per scan. The entire tracking time is 200 scans with a scan period of ΔT=8s, and 100 Monte Carlo runs were performed. The simulations were carried out in MATLAB 2009b on a PC with an Intel(R) Pentium 3.00 GHz CPU and 4 GB RAM.

**Table 1:** The three targets’ parameters.

Target	Target Initial Position (m)	Target Velocity (m/s)
1	(−4000, 2000)	(2, 5)
2	(−3000, 6000)	(2, −5)
3	(0, 6000)	(2, 2)

**Figure 3:**
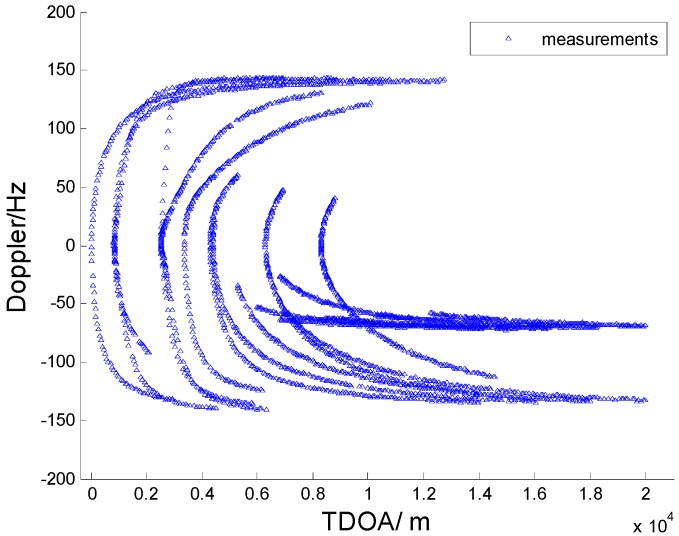
Measurements for three targets without clutter, $\sigma_{p}=0.5$ m, $\sigma_{r}=140$ m, $\sigma_{D}=5$ Hz, λV=0, six transmitters.

**Figure 4:**
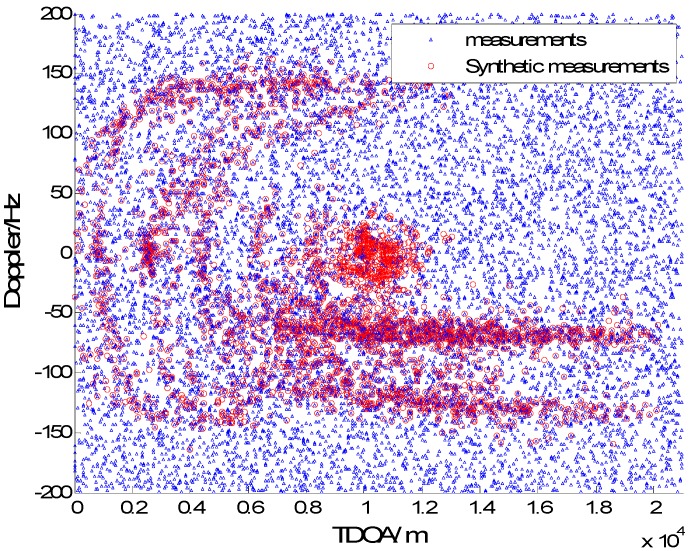
EKF probabilistic multi-hypothesis tracker (PMHTe) synthetic measurements with clutter, $\sigma_{p}=0.5$ m, $\sigma_{r}=140$ m, $\sigma_{D}=5$ Hz, λV=60, six transmitters.

**Figure 5:**
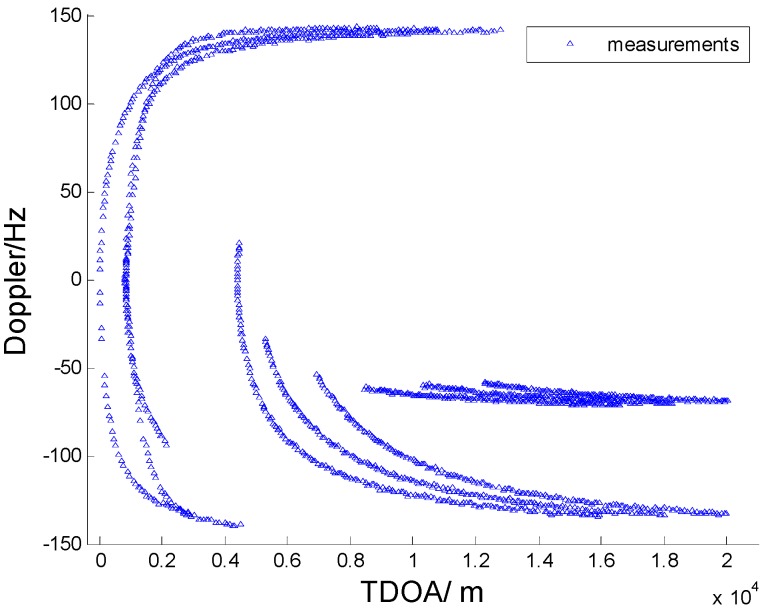
Measurements for three targets without clutter, $\sigma_{p}=0.5$m, $\sigma_{r}=140$m, $\sigma_{D}=5$ Hz, λV=0, three transmitters.

**Figure 6:**
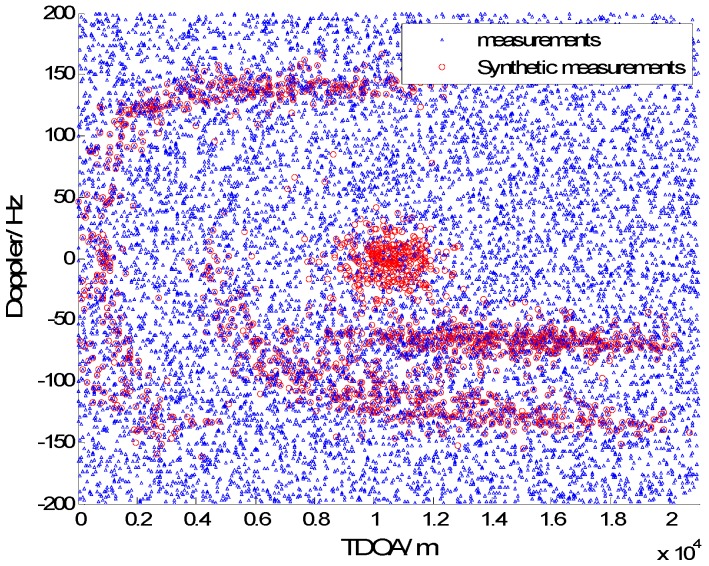
PMHTe synthetic measurements with clutter, $\sigma_{p}=0.5$ m, $\sigma_{r}=140$ m, $\sigma_{D}=5$ Hz, λV=60, three transmitters.

In Figure 3, Figure 4, Figure 5 and Figure 6 we consider the range and Doppler measurements for all targets with and without clutter. Measurements are presented with a blue “triangle”, and synthetic measurements are illustrated with a red “circle”. In Figure 3 and Figure 4, we consider the measurements for three targets with six transmitters on the conditions of without clutter and with clutter. The process noise intensity is $\sigma_{p}=0.5$ m, and the measurement noise for range and Doppler are $\sigma_{r}=140$ m and $\sigma_{D}=5$ Hz. As seen in Figure 4, the clutter is dense, and the synthetic measurements for PMHTe are broadly consistent with the measurements without clutter in Figure 3, which means that the PMHT approach can suppress clutter efficiently. The measurements for three transmitters in the same condition are similar to the case of six transmitters, as seen in Figure 5 and Figure 6.

**Figure 7:**
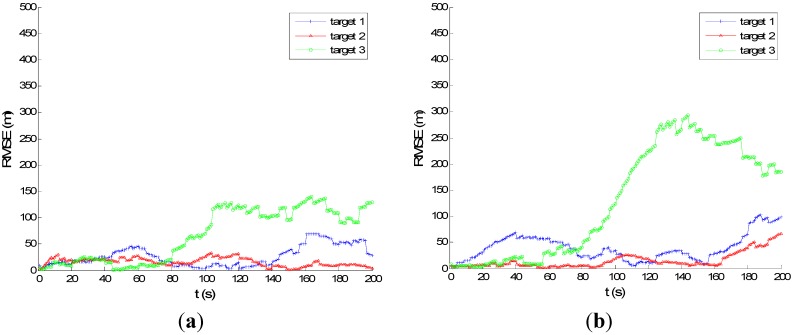
RMSE of position for high measurement noise, $\sigma_{p}=0.5$ m, $\sigma_{r}=140$ m, $\sigma_{\dot{r}}=5$ Hz, λV=60, six transmitters: (**a**) UKF PMHT (PMHTu); (**b**) PMHTe.

**Figure 8:**
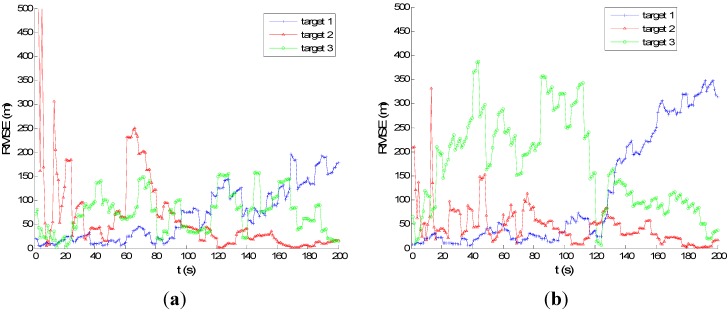
RMSE of position for high measurement noise, $\sigma_{p}=0.5$ m, $\sigma_{r}=140$ m, $\sigma_{D}=5$ Hz, λV=60, three transmitters: (**a**) PMHTu; (**b**) PMHTe.

Figure 7 and Figure 8 show the performance of PMHTe and PMHTu in the sense of root mean square error (RMSE) of position *versus* time scans with a high measurement noise lever for the case of six transmitters and three transmitters, respectively. The measurement noise variances are $\sigma_{r}=140$ m for range and $\sigma_{D}=5$ Hz for Doppler. We can see that for the condition of six transmitters, the PMHTu approach can track all three targets efficiently, and the PMHTe approach works a little worse for Target 3, which is far from the transmitters and receiver. For the condition of three transmitters, the RMSE of position for the PMHTu approach is small for all three targets; while a little worse than the case of six transmitters; the PMHTe method works worse for Targets 1 and 3, as we can see the position errors increase as time elapses. This is because the EKF simply approximates the system function and the measurement function with a first-order Taylor series evaluated at the current estimate of the target state. The EKF ignores higher-order terms and can diverge if it is used in a highly nonlinear system. As opposed to the EKF, the UKF does not approximate the nonlinear measurement function. Instead, it captures the mean and covariance of the target state using deterministic sigma-points based on the unscented transform. The UKF can capture more aspects of the higher order terms, with no Jacobians needed. If the system is highly nonlinear, the EKF may diverge, and the UKF produces typically better results.

**Figure 9:**
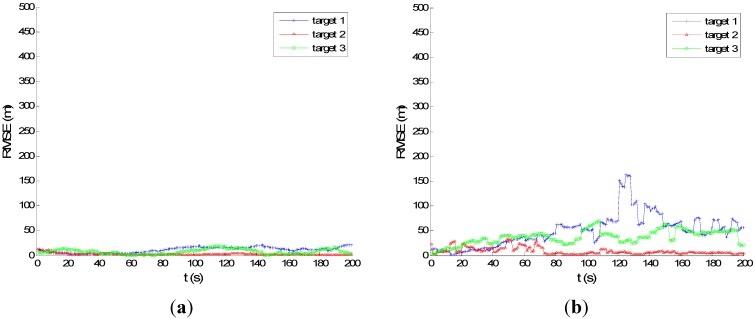
RMSE of position for less challenging situation, $\sigma_{p}=0.5$ m, $\sigma_{r}=70$ m, $\sigma_{D}=1$ Hz, λV=10, $P_{d}=0.8$, six transmitters: (**a**) PMHTu; (**b**) PMHTe.

Now, we consider a less challenging situation for the case of six transmitters, as seen in Figure 9: the process noise intensity is $\sigma_{p}=0.5$ m, and the measurement noise variances are $\sigma_{r}=70$ m for range and $\sigma_{D}=1$ Hz for Doppler. We can see that the position errors for all the three targets are small for both the PMHTe and PMHTu approach. It would appear that the PMHT approach can work adequately in less challenging situations.

## 5. Conclusions

Multi-sensor sonar has many advantages. This paper proposed the EKF-based PMHT and UKF-based PMHT algorithm for the multi-target multi-sensor tracking problem. We employed bistatic range and Doppler measurements. Moreover, except the usual measurement-to-target association, an additional unknown data association between measurements and transmitters is considered in this paper. The simulation results show that for the high measurement noise environment, both the PMHTe and the PMHTu approach work reasonably well when using six transmitters, though PMHTe works a little worse when the target is far from the transmitters and receiver, and the results seems not so good when using three transmitters. However, both PMHTe and PMHTu show better tracking performance in the less challenging environment for six transmitters. Additionally, the run-time complexity is low.

In this work, we used reasonably suitable initial estimates for the PMHT tracker. In the near future, we will focus on the track initialization technique for multistatic tracking and will test the approaches on a real sonar dataset.

## A. Derivation of the Posterior Association Probabilities $w_{m,n}^{\left(l\right)}\left(t,s\right)$

We have:

(A1) $$\begin{array}{ll} p\left(X^{\left(l+1\right)},A,B,Z\right) & =p\left(X^{\left(l+1\right)}\right)p\left(A,B,Z|X^{\left(l+1\right)}\right)\\ & =\prod_{m=1}^{M}p\left(x_{m}^{\left(l+1\right)}\left(1\right)\right)\prod_{t=2}^{T}\prod_{m=1}^{M}p\left(x_{m}^{\left(l+1\right)}\left(t\right)|x_{m}^{\left(l+1\right)}\left(t-1\right)\right)\\ & \;\;\cdot \prod_{s=1}^{S}\prod_{t=1}^{T}\prod_{n=1}^{N_{t}}\pi_{m}^{a}\pi_{s}^{b}p\left(z_{n}\left(t\right)|x_{m}^{\left(l\right)}\left(t\right)\right) \end{array}$$

(A2) $$\begin{array}{ll} p\left(X^{\left(l+1\right)},Z\right)=p & \left(X^{\left(l+1\right)}\right)p\left(Z|X^{\left(l+1\right)}\right)\\ & =\prod_{m=1}^{M}p\left(x_{m}^{\left(l+1\right)}\left(1\right)\right)\prod_{t=2}^{T}\prod_{m=1}^{M}p\left(x_{m}^{\left(l+1\right)}\left(t\right)|x_{m}^{\left(l+1\right)}\left(t-1\right)\right)\\ & \;\;\cdot \prod_{s=1}^{S}\prod_{t=1}^{T}\prod_{n=1}^{N_{t}}\left[\sum_{p=1}^{M}\pi_{p}^{a}\pi_{q}^{b}p\left(z_{n}\left(t\right)|x_{p}^{\left(l\right)}\left(t\right),a_{n}\left(t\right)=p,b_{n}\left(t\right)=q\right)\right] \end{array}$$

We factor:

(A3) $$p\left(A,B\text{|}X^{\left(l\right)}\text{,}Z\right)=\frac{p\left(X^{\left(l\right)}\text{,}A,B,Z\right)}{p\left(X^{\left(l\right)}\text{,}Z\right)}=\prod_{t=1}^{T}\prod_{n=1}^{N}w_{m,n}^{\left(l\right)}\left(t,s\right)$$

From Equations (A1)–(A3), we have:

(A4) $$w_{m,n}^{\left(l\right)}\left(t,s\right)=\frac{\pi_{m}^{a}\pi_{s}^{b}p\left(z_{n}\left(t\right)|x_{m}^{\left(l\right)}\left(t\right),a_{n}\left(t\right)=m,b_{n}\left(t\right)=s\right)}{\sum_{p=1}^{M}\sum_{q=1}^{S}\pi_{p}^{a}\pi_{q}^{b}p\left(z_{n}\left(t\right)|x_{p}^{\left(l\right)}\left(t\right),a_{n}\left(t\right)=p,b_{n}\left(t\right)=q\right)}$$

## B. Derivation of the Function $Q\left(X^{\left(l+1\right)};X^{\left(l\right)}\right)$

From Section 3.2, we know:

(B1) $$Q\left(X^{\left(l+1\right)};X^{\left(l\right)}\right)=\int_{\text{A},\text{B}}\ln\left(p\left(X^{\left(l+1\right)},A,B|Z\right)\right)\cdot p\left(A,B|X^{\left(l\right)}\text{,}Z\right)$$

(B2) $$p\left(A,B\text{|}X^{\left(l\right)}\text{,}Z\right)=\sum_{t=1}^{T}\sum_{n=1}^{N_{t}}w_{m,n}^{\left(l\right)}\left(t,s\right)$$

By the definition of conditional expectation, $p\left(X^{\left(l+1\right)},A,B|Z\right)$ can be written as:

(B3) $$p\left(X^{\left(l+1\right)},A,B|Z\right)=\frac{p\left(X^{\left(l+1\right)},A,B,Z\right)}{p\left(Z\right)}$$

Substituting Equation (B3) into Equation (B1), we get:

(B4) $$Q\left(X^{\left(l+1\right)};X^{\left(l\right)}\right)=\int_{\text{A},\text{B}}\ln\left(p\left(X^{\left(l+1\right)},A,B,Z\right)\right)p\left(A,B|X^{\left(l\right)}\text{,}Z\right)-\ln p\left(Z\right)$$

Since lnp(Z) is a constant, it can be dropped from Equation (B4). Additionally, we have:

(B5) $$\begin{array}{ll} p\left(X^{\left(l+1\right)},A,B,Z\right) & =p\left(X^{\left(l+1\right)}\right)p\left(A,B,Z|X^{\left(l+1\right)}\right)\\ & =\prod_{m=1}^{M}p\left(x_{m}^{\left(l+1\right)}\left(1\right)\right)\prod_{t=2}^{T}\prod_{m=1}^{M}p\left(x_{m}^{\left(l+1\right)}\left(t\right)|x_{m}^{\left(l+1\right)}\left(t-1\right)\right)\\ & \;\;\cdot \prod_{s=1}^{S}\prod_{t=1}^{T}\prod_{n=1}^{N_{t}}\pi_{m}^{a}\pi_{s}^{b}p\left(z_{n}\left(t\right)|x_{m}^{\left(l\right)}\left(t\right),a_{n}\left(t\right)=m,b_{n}\left(t\right)=s\right) \end{array}$$

From Equations (B1), (B2), (B4) and (B5), we get:

(B6) $$\begin{array}{l} Q\left(X^{\left(l+1\right)};X^{\left(l\right)}\right)=\sum_{A,B}\ln\left(p\left(X^{\left(l+1\right)},A,B\text{|}Z\right)\right)p\left(A,B|X^{\left(l\right)},Z\right)\\ =\ln\left(\prod_{m=1}^{M}p\left(x_{m}^{\left(l+1\right)}\left(1\right)\right)\prod_{t=2}^{T}\prod_{m=1}^{M}p\left(x_{m}^{\left(l+1\right)}\left(t\right)|x_{m}^{\left(l+1\right)}\left(t-1\right)\right)\right)\\ +\sum_{s=1}^{S}\sum_{t=1}^{T}\sum_{n=1}^{N_{t}}\sum_{m=1}^{M}(w_{m,n}^{\left(l\right)}\left(t,s\right)\log\left(\pi_{m}^{a}\pi_{s}^{b}\right)+w_{m,n}^{\left(l\right)}\left(t,s\right)\ln p\left(z_{n}\left(t\right)|x_{m}^{\left(l\right)}\left(t\right),a_{n}\left(t\right)=m,b_{n}\left(t\right)=s\right)) \end{array}$$
